# Environmental dust inhalation in the European badger (*Meles meles*): Systemic distribution of silica-laden macrophages, pathological changes, and association with *Mycobacterium bovis* infection status

**DOI:** 10.1371/journal.pone.0190230

**Published:** 2018-01-17

**Authors:** Janne M. Schoening, Leigh A. L. Corner, Locksley L. McV. Messam, Joseph P. Cassidy, Alan Wolfe

**Affiliations:** School of Veterinary Medicine, University College Dublin (UCD), Dublin, Ireland; Animal and Plant Health Agency, UNITED KINGDOM

## Abstract

Chronic inhalation of crystalline silica and silicates may lead to severe lung disease in humans, termed silicosis. The disease is an occupational health concern in miners and related professions worldwide. Silicosis is also a strong risk factor for tuberculosis in humans. Due to its subterranean lifestyle, the European badger (*Meles meles*) is continuously exposed to environmental dust, while this species is also susceptible to tuberculosis, caused by *Mycobacterium bovis*. To date, a thorough investigation of mineral dust retention and its possible implication as a risk factor for mycobacterial infection in badgers has not been performed. The aims of this retrospective histological study were (1) to describe the systemic tissue distribution of silica-laden macrophages (SLMs) in badgers; (2) to compare the amount of SLMs in tissues of badgers of differing *M*. *bovis* infection status, pulmonary SLM burden and age; and (3) to assess whether inflammation was associated with SLMs. We assessed lung, lymph nodes, liver and spleen of 60 wild-caught badgers of known *M*. *bovis* infection status for the presence of SLMs using polarizing light microscopy. SLMs were consistently present within the lungs and were widely distributed throughout the lymphatic system. No inflammatory reaction to SLMs, as occurs in human silicosis, was observed in any tissue. Distribution and amount of SLMs were similar between *M*. *bovis* positive and negative badgers, and we were not able to show an association between the amount of SLMs and *M*. *bovis* infection status. The amount of SLMs within intra- and extrathoracic lymph nodes was positively associated with the amount of pulmonary SLMs, and with age. This is the first report of substantial and systemic tissue retention of mineral dust particles in a mammalian species lacking associated chronic inflammation (i.e. silicosis). We further highlight different pathogenetic mechanisms underlying silicosis and benign SLM accumulations following siliceous dust inhalation.

## Introduction

Silicon dioxide (SiO_2_), in its crystalline form alone (silica) or in combination with various trace elements, e.g. Al, Fe, Mg and others (silicates), constitutes a wide variety of rocks and sands worldwide, and is a major component of inorganic dusts [1].

Finely ground and aerosolized silica and silicates pose an occupational health hazard to mine workers, sandblasters and related professions, where persistent inhalation leads to silicosis, a chronic-progressive fibrosing lung disease [2, 3]. Affected organs include the lungs and draining lymph nodes [4], while more systemic, extrathoracic lesions are rare [5–7].

Crystalline silica is toxic to alveolar macrophages, leading to the formation of reactive oxygen species among other cytotoxic effects [8–11]. The release of various proinflammatory and profibrotic factors results in sustained inflammation, and ultimately fibrosis that compromises respiratory function [12, 13]. Silicosis, and also silica inhalation in the absence of chronic disease, are strong risk factors for tuberculosis in miners [14–17]. While the exact underlying pathogenesis of this relationship remains unclear, modulatory effects of silica on pulmonary immune function, including macrophage activity, have been suggested [18, 19].

Granulomatous inflammation centred on silica-laden macrophages (SLMs) resembling silicosis in humans has been reported in other species, including horses, dogs, and otters [20–22]. In contrast, accumulations of SLMs with mild or no associated inflammation or clinical lung disease have occasionally been described in avian and mammalian species, including humans, from arid regions [23–26].

The European badger (*Meles meles*) is naturally and continuously exposed to environmental dust due to its subterranean lifestyle, burrowing and foraging habits. Although SLMs are found within their lungs and intrathoracic lymph nodes [27–29], the distribution of these cells and any potential associated inflammatory response has not been studied in detail in this species. Of further relevance is the fact that the badger can develop both clinical and subclinical tuberculosis and act as a maintenance host for *Mycobacterium (M*.*) bovis*, the main causative agent of tuberculosis in cattle [30, 31]. An immune-suppressive effect of inhaled silica, similar to that proposed in humans, has been suggested to render the badger more susceptible to *M*. *bovis* infection [28].

As part of a previous study of *M*. *bovis* infection in wild-caught badgers, we assessed a wide range of lung and lymphatic tissues histopathologically for the presence of tuberculous lesions [32]. During these analyses, macrophages containing crystalline material, consistent with SLMs, were frequently noted in the lungs and intrathoracic lymph nodes, and a brief investigation into this additional finding was done. Preliminary analyses showed no difference in the amount of crystalline particles within the lungs between *M*. *bovis* infected and uninfected individuals [32]. Also observed in that study, but not reported, was the presence of SLMs in lymphatic tissues outside the thorax (L. Corner, personal communication). The present study was subsequently designed to examine these preliminary observations more rigorously using an objective SLM scoring system, and expanding the investigation to include extrathoracic lymphatic tissues, liver and spleen. Specifically, our key objectives were: (1) to fully describe the systemic distribution of SLMs in a wide range of badger tissues; (2) to compare the distribution and quantity of SLMs in tissues of badgers of differing *M*. *bovis* infection status, pulmonary SLM burden and age. An additional objective was to assess if SLM accumulation within tissues is associated with chronic inflammation.

## Materials and methods

No animals were killed for the specific purpose of this investigation. We conducted all quantitative examinations (SLM scoring) on archived histological slides from a previous study, for which all badgers were wild caught and legally culled under license from the National Parks and Wildlife Service of the Department of the Environment, Heritage and Local Government [32].

Additional qualitative investigations were performed using tissues from wild caught badgers from a separate study, for which badgers were euthanized under a research study licence (AE19113/P003) issued by the Health Products Regulatory Authority (HPRA) and approved by the UCD Animal Research Ethics Committee (AREC): AREC-14-06-Gormley. For handling, badgers were anaesthetised with an intramuscular injection of ketamine hydrochloride (10 mg/kg) and medetomidine hydrochloride (0.1 mg/kg, Domitor®, Pfizer). Anaesthetised badgers were euthanized with an intravenous overdose of sodium pentobarbital.

### Quantitative investigations

#### Materials

We examined the following tissues from 60 badgers for the presence of SLMs: mandibular lymph nodes, parotid lymph nodes, tonsils, retropharyngeal lymph nodes, prescapular lymph nodes, axillary lymph nodes, tracheobronchial lymph nodes, anterior mediastinal lymph nodes, posterior mediastinal lymph nodes, inguinal lymph nodes, popliteal lymph nodes, mesenteric lymph nodes, hepatic lymph nodes, lung, liver, and spleen. Where organs and lymph nodes occurred in pairs, each was examined separately and each lung lobe was examined individually. All tissues were embedded in paraffin, processed as 3 μm sections and routinely stained with hematoxylin and eosin for histological examination.

The archive from which materials were sourced contained slide sets of 132 wild-caught badgers, comprising 57 *M*. *bovis* positive and 75 *M*. *bovis* negative animals. Of these, 30. *M*. *bovis* positive, and 30. *M*. *bovis* negative individuals, respectively, were randomly chosen. If intra-pulmonary SLMs were absent in any given individual, this badger was excluded, and the next randomly chosen badger with intra-pulmonary SLMs was included. The omission of individuals lacking SLMs within their lungs, which applied to single cases only, was done to exclude individuals, which may possibly not have been exposed to environmental dust inhalation for any reason. Further details on age, geographic origin, and sex of *M*. *bovis* positive and negative badgers are available in S2 Table. Age estimation was based on body weight and the degree of dental attrition, and badgers were grouped into three age classes, juvenile, adult, and old, accordingly [32]. Positive and negative *M*. *bovis* infection status was determined by mycobacterial culture [32]. Of the 30 *M*. *bovis* culture positive badgers in this study with 2–30 culture positive tissues/badger (mean: 7.9 tissues/animal), 22 badgers also had granulomatous lesions containing acid-fast bacilli in 1–28 tissues (mean: 4.7 tissues/animal), and 17 individuals had gross tuberculous lesions in 1–15 tissues/badger (mean: 2.6 tissues/animal).

#### Microscopic assessment

**Fig 1:**
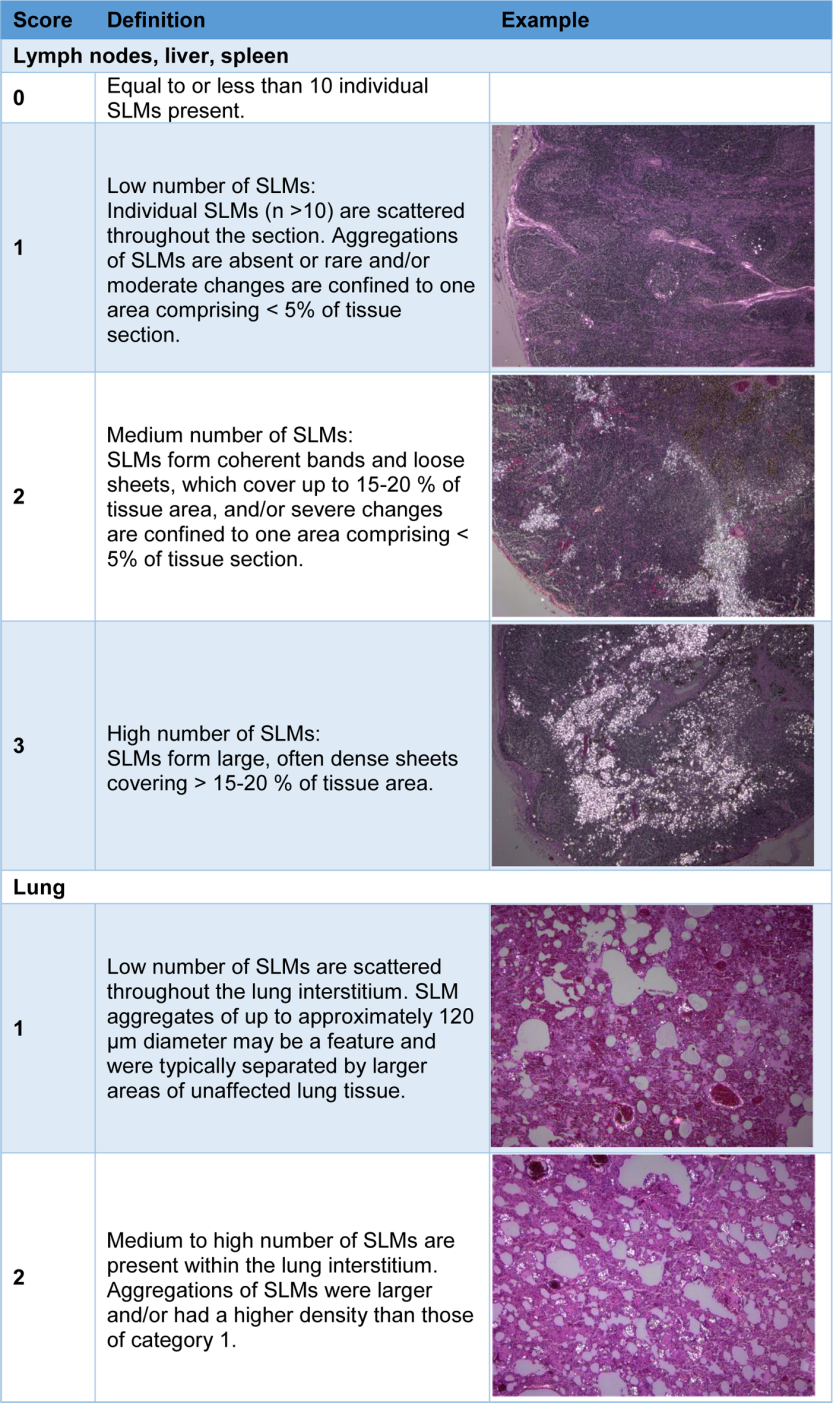
Scoring categories used for assessing the number of silica-laden macrophages (SLMs) within badger tissues. Under polarized light, SLMs contrast brightly against the darker background tissue. For a higher magnification view of SLMs, see also Figs 2 and 3.

**Fig 2:**
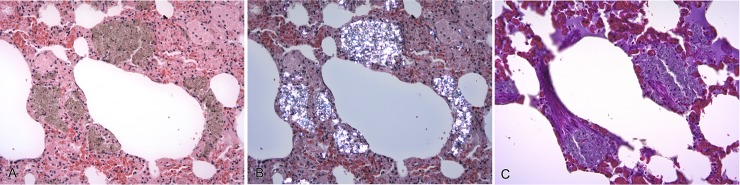
Silica-laden macrophages (SLMs), lung, badger. Multifocal interstitial accumulations of macrophages engorged with crystalline material. (A) Crystalline material has a light brown color and finely granular to spicular appearance in routinely stained sections. H&E. x200. (B) Crystalline material is strongly birefringent under polarizing light, consistent with silica and silicates. H&E, x200. (C) No evidence of associated collagen deposition on Massons trichrome stain (stains collagen blue). x200.

**Fig 3:**
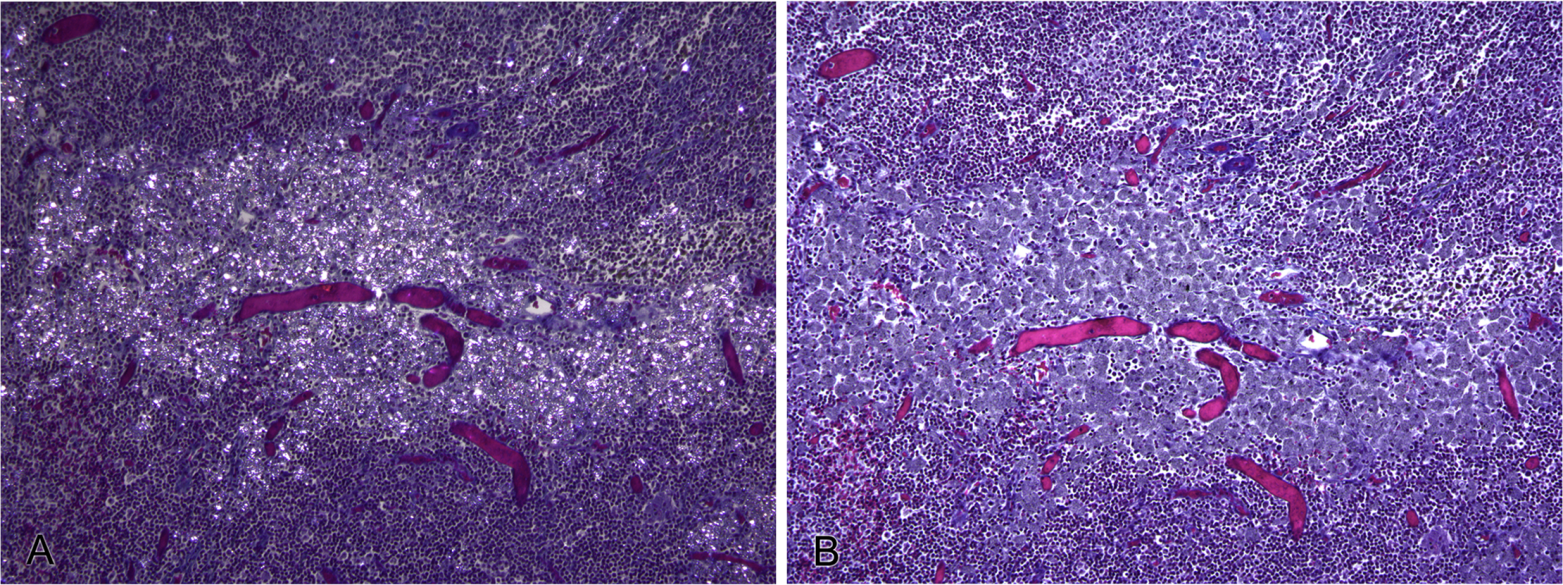
Silica-laden macrophages (SLMs), bronchial lymph node, badger. Focally-extensive aggregation of SLMs in the absence of collagen deposition. (A) SLMs are strongly birefringent under polarizing light. Massons trichrome stain. x100. (B) No evidence of associated collagen deposition on Massons trichrome stain (stains collagen blue). x100.

We examined all tissues at 40x, 100x and/or 200x magnification for the presence of SLMs under polarized light using an Olympus BX 51 microscope with accessory polarizer. A SLM was defined as an engorged macrophage, measuring 20–40 μm in diameter (occasionally up to 100 μm), with light brown cytoplasm, containing one or more birefringent particles of varying size consistent with silica and silicates, as previously described [21, 22, 24, 28, 33]. We categorized the amount of SLMs within lung tissue as either ‘1’ (low number of SLMs) or ‘2’ (high number of SLMs) (Fig 1). We assessed the amount of SLMs in lymph node, liver and spleen tissue after defining and validating a semi-quantitative scoring system ranging from ‘0’ (equal to or less than 10 SLMs) to ‘3’ (high number of SLMs) (Fig 1; for a higher magnification view of SLMs, see also Figs 2 and 3). For each lymphoid tissue, slides contained between 1 and 9 sections of the same lymphoid tissue or lymph center. When more than one section of tissue was present on a slide, all sections were assessed as one area.

In 53/60 badgers the same SLM score was recorded in each of their six lung lobes. In the remaining seven badgers at least four out of six lung lobes had the same SLM score. Therefore, in each animal the overall predominant lung SLM score was used for statistical analysis.

To investigate differences in the number of SLMs between tissues, cumulative SLM scores (representing the total sum of SLM scores across all 60 individuals) were calculated for each tissue. Note was taken for any tissues, of inflammatory infiltrates or excess fibrous tissue in association with SLM accumulations.

#### Validation of scoring system

We validated lymph node SLM scores on 30 slides (10 slides each for scoring categories 1–3) by placing each slide under an orthogonal grid (10x10 dots). Ten dots were selected using a random number generator (www.random.org). The tissue area immediately adjacent to each selected dot was then examined at 200x magnification using another 10x10 grid (10mm^2^ micrometer eyepiece). One hundred fields were thus examined for each of the 10 randomly selected dots per slide, and the number of positive fields recorded. A positive field was one that contained one or more SLMs. In total, 1000 fields for each slide were therefore examined.

All lung SLM scores were re-evaluated by the same investigator (JS) three to six months after initial assessment and the intra-observer agreement was calculated.

#### Statistical analysis

Cumulative SLM scores per tissue, and graphs describing the distribution and quantity of SLMs between different tissues, as well as for badgers of differing *M*. *bovis* infection status and pulmonary SLM burden, were created using Microsoft Excel 2013. Inferential statistical analyses were performed using SPSS Statistics Software Package, version 20. The Mann-Whitney U Test was used to compare the distribution of SLM scores in different tissues between two independent groups of badgers. Groups were defined by either *M*. *bovis* infection status (*M*. *bovis* positive animals: n = 30 vs. *M*. *bovis* negative animals: n = 30) or lung SLM score (score 1: n = 33 vs. score 2: n = 27). Differences in SLM burden in lymphatic tissues between age groups were evaluated for statistical significance using the Kruskall-Wallis test. For lung tissue, the Chi Square test was used to examine differences in SLM burden between badgers of differing *M*. *bovis* infection status, as well as between age groups. Tissues that contained no or few SLMs were excluded from these analyses. Intra-observer agreement of lung SLM scores was calculated using Cohens kappa.

For all calculations, p ≤ 0.05 was considered statistically significant.

### Qualitative investigations

#### Materials

Paraffin blocks of tissues assessed for SLM scoring were not available. Therefore, further qualitative investigations were performed on seven wild-caught badgers from a separate study, for which such tissue material was available. These badgers had similar SLM burdens within their lungs and other tissues as the individuals examined for the quantitative part of the study.

#### Microscopic assessment

To confirm the absence of excess fibrous tissue or collagen associated with SLM accumulations, Massons trichrome stains were performed on a total of four sections of lung tissue from four individual badgers, using two sections of each scoring category 1 and 2. Massons trichrome stains were also performed on a total of three bronchial lymph nodes from two badgers, comprising one section from each of the scoring categories 1–3.

Further, during quantitative assessment of lymph node tissues, extrathoracic peaks in SLM score were observed within the axillary and popliteal lymph nodes, which drain the front and hind limbs, respectively. Therefore, we wanted to investigate a possible percutaneous route of silica and silicate entry. For this purpose, we evaluated grossly unremarkable foot pad sections of four badgers for the presence of SLMs using polarizing light microscopy as detailed above.

## Results

### Distribution of SLMs

**Fig 4:**
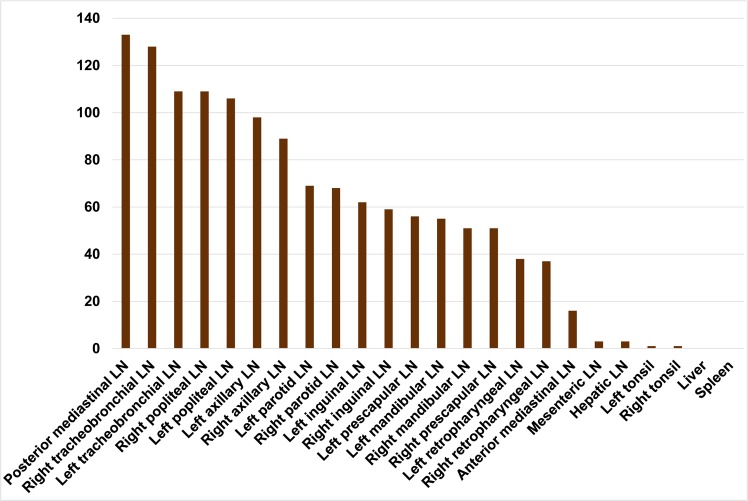
Distribution and cumulative silica-laden macrophage (SLM) score across badger lymph nodes, tonsils, liver and spleen. Bars represent the cumulative SLM score per tissue across the study population of 60 badgers.

SLMs were widely distributed throughout the lungs and lymphatic system. Intrathoracic lymph nodes, namely tracheobronchial and mediastinal lymph nodes, showed the highest amount of SLMs, with the exception of the anterior mediastinal lymph node (Fig 4). High SLM scores were also observed in the axillary and popliteal lymph nodes. The following organs showed a frequent absence or only low number of SLMs: Liver (0/60 with SLMs), spleen (0/60), hepatic lymph node (3/60), mesenteric lymph node (3/60), left tonsil (1/60), right tonsil (1/60) and anterior mediastinal lymph node (14/60). No inflammatory response or excess fibrous tissue associated with the presence of SLMs was microscopically detectable in any of these routinely stained sections (Fig 2A and 2B).

### Tissue SLM score and *M*. *bovis* infection status

**Fig 5:**
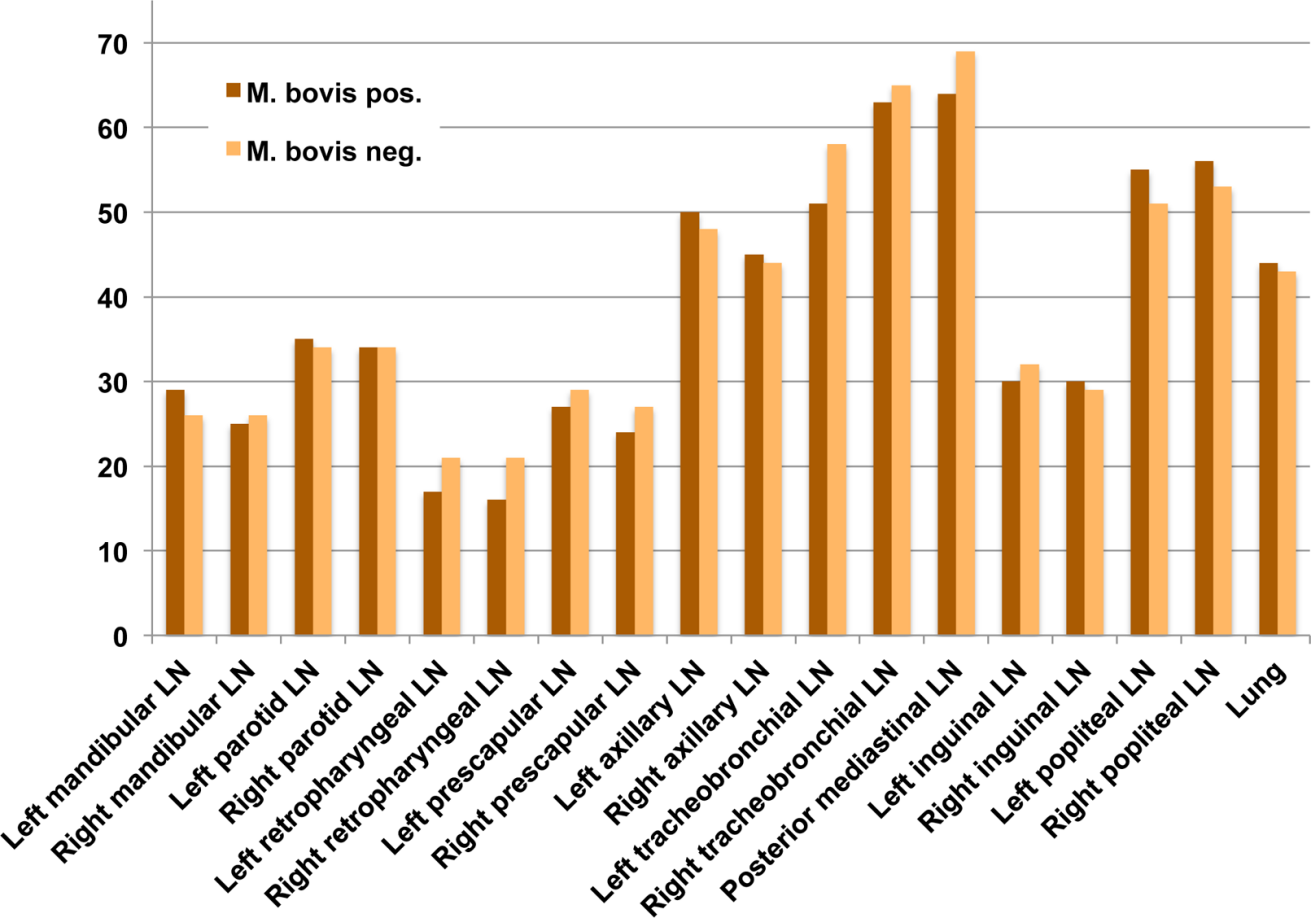
Bar chart comparing cumulative silica-laden macrophage (SLM) scores in tissues from badgers with positive and negative *M*. *bovis* infection status. Bars represent the cumulative SLM score per tissue across groups of *M*. *bovis* positive (n = 30) and *M*. *bovis* negative (n = 30) individuals.

*M*. *bovis* positive and negative animals had similar quantities and a similar distribution of SLMs across all tissues examined (Fig 5). Differences in SLM distribution were statistically not significant (Table 1).

**Table 1:** Distribution of silica-laden macrophage (SLM) scores in lymph nodes and lung tissue of *M*. *bovis* positive and negative badgers.

Tissue	*M*. *bovis* infection status	SLM score* n (%)				pValue^MWT^
		0	1	2	3	
**Left mandibular LN**	pos	6 (20.0)	19 (63.3)	5 (16.7)	-	0.52
	neg	6 (20.0)	22 (73.3)	2 (6.7)	-	
**Right mandibular LN**	pos	8 (26.7)	19 (63.3)	3 (10.0)	-	0.77
	neg	6 (20.0)	22 (73.3)	2 (6.7)	-	
**Left parotid LN**	pos	-	25 (83.3)	5 (16.7)	-	0.72
	neg	-	26 (86.7)	4 (13.3)	-	
**Right parotid LN**	pos	2 (6.7)	22 (73.3)	6 (20.0)	-	0.97
	neg	1 (3.3)	24 (80.0)	5 (16.7)	-	
**Left retropharyngeal LN**	pos	13 (43.3)	17 (56.7)	-	-	0.29
	neg	9 (30.0)	21 (70.0)	-	-	
**Right retropharyngeal LN**	pos	14 (46.7)	16 (53.3)	-	-	0.19
	neg	9 (30.0)	21 (70.0)	-	-	
**Left prescapular LN**	pos	7 (23.3)	19 (63.3)	4 (13.3)	-	0.58
	neg	3 (10.0)	25 (83.3)	2 (6.7)	-	
**Right prescapular LN**	pos	9 (30.0)	18 (60.0)	3 (10.0)	-	0.38
	neg	4 (13.3)	25 (83.3)	1 (3.3)	-	
**Left axillary LN**	pos	-	13 (43.3)	14 (46.7)	3 (10.0)	0.60
	neg	-	16 (53.3)	10 (33.3)	4 (13.3)	
**Right axillary LN**	pos	-	17 (56.7)	11 (36.7)	2 (6.7)	0.71
	neg	-	19 (63.3)	8 (26.7)	3 (10.0)	
**Left inguinal LN**	pos	2 (6.7)	26 (86.7)	2 (6.7)	-	0.52
	neg	2 (6.7)	24 (80.0)	4 (13.3)	-	
**Right inguinal LN**	pos	3 (10.0)	24 (80.0)	3 (10.0)	-	0.72
	neg	1 (3.3)	29 (96.7)	-	-	
**Left popliteal LN**	pos	-	12 (40.0)	11 (36.7)	7 (23.3)	0.54
	neg	-	13 (43.3)	13 (43.3)	4 (13.3)	
Right popliteal LN**	pos	-	10 (34.5)	11 (37.9)	8 (27.6)	0.43
	neg	-	12 (40.0)	13 (43.3)	5 (16.7)	
**Left tracheobronchial LN**	pos	2 (6.7)	14 (46.7)	5 (16.7)	9 (30.0)	0.26
	neg	1 (3.3)	6 (20.0)	17 (56.7)	6 (20.0)	
**Right tracheobronchial LN**	pos	-	8 (26.7)	11 (36.7)	11 (36.7)	0.80
	neg	-	4 (13.3)	17 (56.7)	9 (30.0)	
**Posterior mediastinal LN**	pos	-	9 (30.0)	8 (26.7)	13 (43.3)	0.52
	neg	-	3 (10.0)	15 (50.0)	12 (40.0)	
**Lung**	pos	-	16 (53.3)	14 (46.7)	-	0.80^chi^
	neg	-	17 (56.7)	13 (43.3)	-	

Badgers with *M*. *bovis* positive and negative infection status did not show significant differences in the distribution of SLM scores across lymphatic and lung tissues.

*for lymph nodes (LN), SLM scores 0; 1; 2; and 3 correspond to no SLMs; low; medium; and high SLM burden, respectively. For lung tissue, SLM scores 1 and 2 correspond to low and high SLM burden, respectively.

^MWT^ = all pValues were calculated using the Mann-Whitney U test for comparing the two groups of badgers with *M*. *bovis* positive (n = 30, except **n = 29) and negative (n = 30) infection status, except ^chi^: χ^2^-test. Pos = positive. Neg = negative. “-”= score not observed.

### Lung SLM score and lymph node SLM score

**Fig 6:**
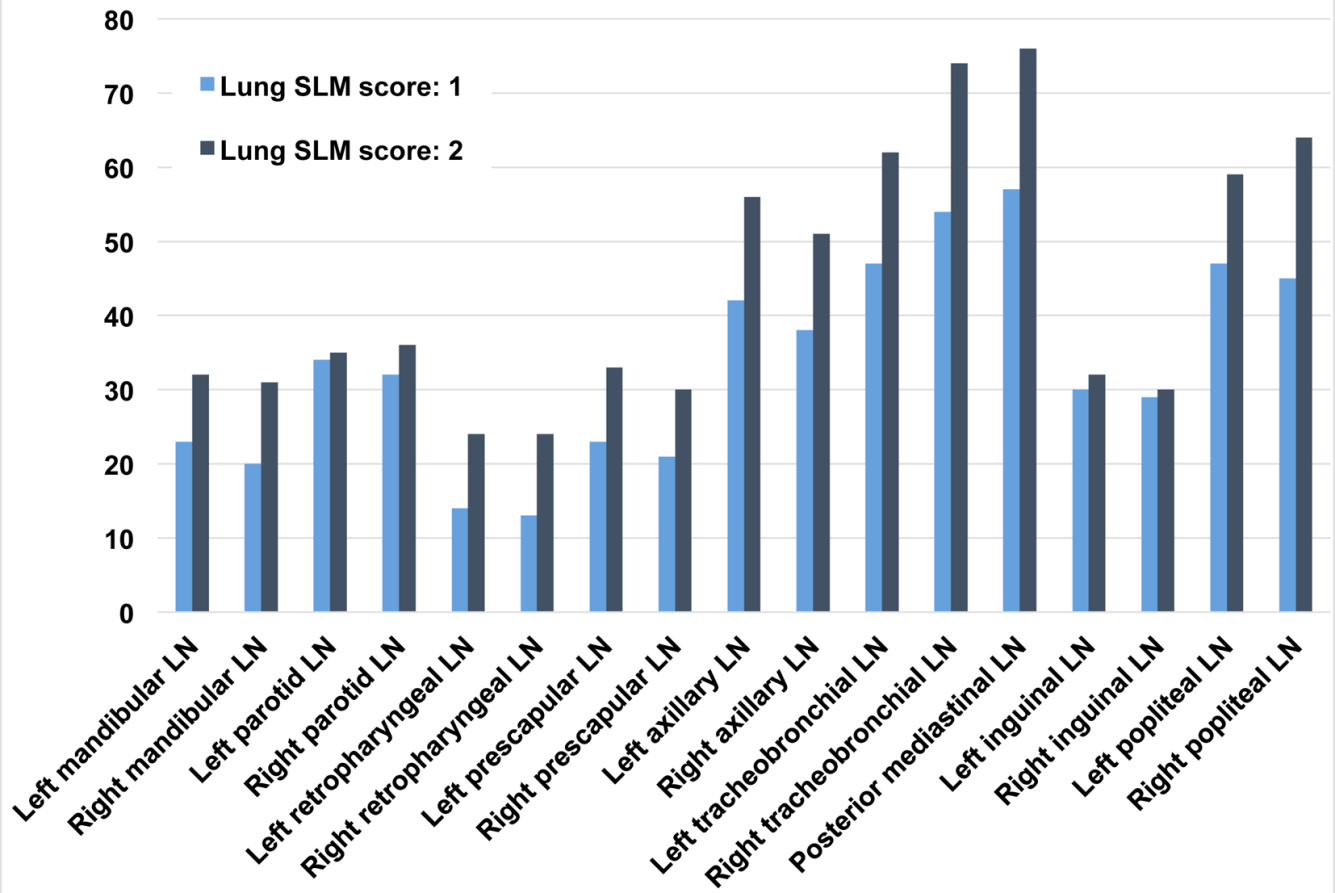
Bar chart comparing cumulative silica-laden macrophage (SLM) scores in tissues from badgers with low and high SLM lung scores. Bars represent the cumulative SLM score per tissue across groups of individuals with SLM lung scores of either ‘1’ (n = 33) or ‘2’ (n = 27).

Lymph node SLM scores were positively associated with lung SLM scores. Badgers with a high lung SLM score (‘2’) had a higher overall SLM score (Fig 6), as well as a higher frequency of medium and high lymph node SLM scores, within all intra- and extrathoracic tissues, compared to those with a low lung SLM score (‘1’). These differences in SLM distribution were statistically significant across all tissues analysed (Table 2).

**Table 2:** Distribution of silica-laden macrophage (SLM) scores in lymph nodes of badgers with a high and low lung SLM score, respectively.

Tissue	Lung SLM score	SLM score* n (%)				pValue^MWT^
		0	1	2	3	
**Left mandibular LN**	high	1 (3.7)	20 (74.1)	6 (22.2)	-	0.001
	low	11 (33.3)	21 (63.3)	1 (3.0)	-	
**Right mandibular LN**	high	1 (3.7)	21 (77.8)	5 (18.5)	-	<0.001
	low	13 (39.4)	20 (60.6)	-	-	
**Left parotid LN**	high	-	19 (70.4)	8 (29.6)	-	0.004
	low	-	32 (97.0)	1 (3.0)	-	
**Right parotid LN**	high	1 (3.7)	16 (59.3)	10 (37.0)	-	0.002
	low	2 (6.1)	30 (90.9)	1 (3.0)	-	
**Left retropharyngeal LN**	high	3 (11.1)	24 (88.9)	-	-	<0.001
	low	19 (57.6)	14 (42.4)	-	-	
**Right retropharyngeal LN**	high	3 (11.1)	24 (88.9)	-	-	<0.001
	low	20 (60.6)	13 (39.4)	-	-	
**Left prescapular LN**	high	-	21 (77.8)	6 (22.2)	-	<0.001
	low	10 (30.3)	23 (69.7)	-	-	
**Right prescapular LN**	high	1 (3.7)	22 (81.5)	4 (14.8)	-	<0.001
	low	12 (36.4)	21 (63.6)	-	-	
**Left axillary LN**	high	-	5 (18.5)	15 (55.6)	7 (25.9)	<0.001
	low	-	24 (72.7)	9 (27.3)	-	
**Right axillary LN**	high	-	8 (29.6)	14 (51.9)	5 (18.5)	<0.001
	low	-	28 (84.8)	5 (15.2)	-	
**Left inguinal LN**	high	-	22 (81.5)	5 (18.5)	-	0.01
	low	4 (12.1)	28 (84.4)	1 (3.0)	-	
**Right inguinal LN**	high	-	24 (88.9)	3 (11.1)	-	0.009
	low	4 (12.1)	29 (87.9)	-	-	
**Left popliteal LN**	high	-	5 (18.5)	12 (44.4)	10 (37.0)	<0.001
	low	-	20 (60.6)	12 (36.4)	1 (3.0)	
**Right popliteal LN**	high	-	2 (7.4)	13 (48.1)	12 (44.4)	<0.001
	low	-	20 (62.5)	11 (34.4)	1 (3.1)	
**Left tracheobronchial LN**	high	1 (3.7)	4 (14.8)	8 (29.6)	14 (51.9)	<0.001
	low	2 (6.1)	16 (48.5)	14 (42.4)	1 (3.0)	
**Right tracheobronchial LN**	high	-	-	7 (25.9)	20 (74.1)	<0.001
	low	-	12 (36.4)	21 (63.6)	-	
**Posterior mediastinal LN**	high	-	1 (3.7)	3 (11.1)	23 (85.2)	<0.001
	low	-	11 (33.3)	20 (60.6)	2 (6.1)	

Badgers with a high lung SLM score also had significantly higher SLM scores across lymph nodes compared to badgers with a low lung SLM score.

*SLM scores 0; 1; 2; and 3 correspond to no SLMs; low; medium; and high SLM burden, respectively.

^MWT^ = all pValues calculated using the Mann-Whitney U test for comparing the two groups of badgers with high (n = 27) and low (n = 33) lung SLM score. LN = lymph node. “-”= score not observed

### Tissue SLM score and age

**Fig 7:**
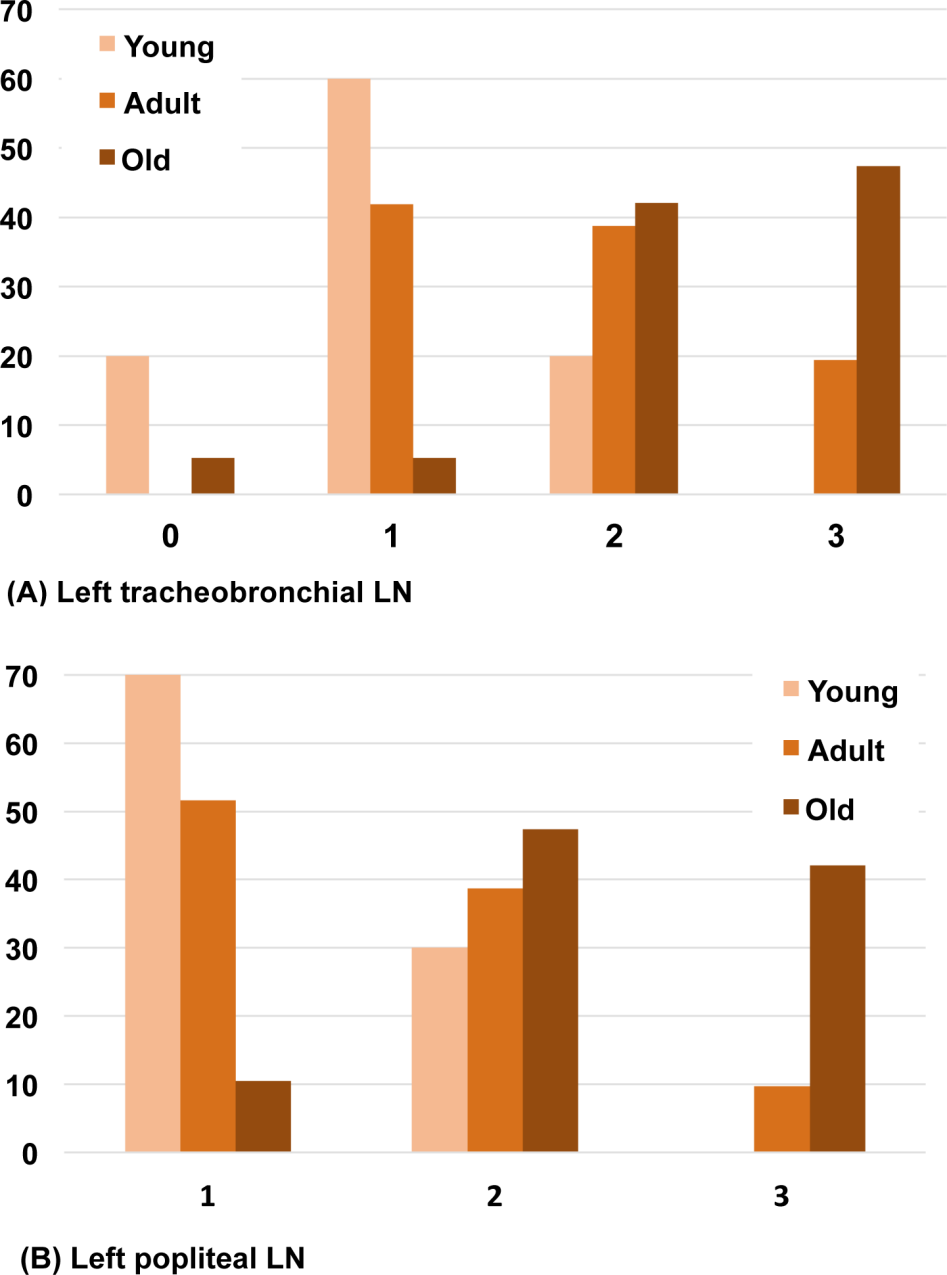
Grouped bar graph illustrating the proportion (in %) of badgers in each scoring category (x-axis) among age classes. Given are examples of score distribution among age classes (young (n = 10); adult (n = 31); old (n = 19)) for an intrathoracic (A) and extrathoracic (B) lymph node (LN). (A) Left tracheobronchial LN; (B) Left popliteal LN. Differences in distribution between age classes were statistically significant (Table 3).

The distribution of tissue SLM scores differed significantly between age classes and SLM score was positively associated with animal age. Old badgers had higher SLM scores in their lungs, and in intra- and extrathoracic lymph nodes, than both adult and young animals (Table 3, Fig 7).

**Table 3:** The distribution of silica-laden macrophage scores in badger lymphatic tissues and lung among age classes.

Tissue	Score	Age			pValue^KWT^
		Young	Adult	Old	
		n (%)	n (%)	n (%)	
**Left mandibular LN**	0	5 (50)	6 (19.4)	1 (5.3)	0.004
	1	5 (50)	23 (74.2)	13 (68.4)	
	2	0 (0)	2 (6.5)	5 (26.3)	
	3	0 (0)	0 (0)	0 (0)	
**Right mandibular LN**	0	6 (60)	8 (25.8)	0 (0)	<0.001
	1	4 (40)	22 (71)	15 (78.9)	
	2	0 (0)	1 (3.2)	4 (21.1)	
	3	0 (0)	0 (0)	0 (0)	
**Left parotid LN**	0	0 (0)	0 (0)	0 (0)	0.005
	1	10 (100)	29 (93.5)	12 (63.2)	
	2	0 (0)	2 (6.5)	7 (36.8)	
	3	0 (0)	0 (0)	0 (0)	
**Right parotid LN**	0	0 (0)	2 (6.5)	1 (5.3)	0.022
	1	10 (100)	26 (83.9)	10 (52.6)	
	2	0 (0)	3 (9.7)	8 (42.1)	
	3	0 (0)	0 (0)	0 (0)	
**Left retropharyngeal LN**	0	7 (70)	13 (41.9)	2 (10.5)	0.005
	1	3 (30)	18 (58.1)	17 (89.5)	
	2	0 (0)	0 (0)	0 (0)	
	3	0 (0)	0 (0)	0 (0)	
**Right retropharyngeal LN**	0	7 (70)	14 (45.2)	2 (10.5)	0.004
	1	3 (30)	17(54.8)	17 (89.5)	
	2	0 (0)	0 (0)	0 (0)	
	3	0 (0)	0 (0)	0 (0)	
**Left prescapular LN**	0	5 (50)	5 (16.1)	0 (0)	0.002
	1	5 (50)	24 (77.4)	15 (78.9)	
	2	0 (0)	2 (6.5)	4 (21.1)	
	3	0 (0)	0 (0)	0 (0)	
**Right prescapular LN**	0	5 (50)	8 (25.8)	0 (0)	0.007
	1	5 (50)	21 (67.7)	17 (89.5)	
	2	0 (0)	2 (6.5)	2 (10.5)	
	3	0 (0)	0 (0)	0 (0)	
**Left axillary LN**	0	0 (0)	0 (0)	0 (0)	<0.001
	1	9 (90)	16 (51.6)	4 (21.1)	
	2	1 (10)	14 (45.2)	9 (47.4)	
	3	0 (0)	1 (3.2)	6 (31.6)	
**Right axillary LN**	0	0 (0)	0 (0)	0 (0)	0.004
	1	8 (80)	22 (71)	6 (31.6)	
	2	2 (20)	8 (25.8)	9 (47.4)	
	3	0 (0)	1 (3.2)	4 (21.1)	
**Left tracheobronchial LN**	0	2 (20)	0 (0)	1 (5.3)	<0.001
	1	6 (60)	13 (41.9)	1 (5.3)	
	2	2 (20)	12 (38.7)	8 (42.1)	
	3	0 (0)	6 (19.4)	9 (47.4)	
**Right tracheobronchial LN**	0	0 (0)	0 (0)	0 (0)	<0.001
	1	7 (70)	5 (16.1)	0 (0)	
	2	3 (30)	19 (61.3)	6 (31.6)	
	3	0 (0)	7 (22.6)	13 (68.4)	
**Posterior mediastinal LN**	0	0 (0)	0 (0)	0 (0)	<0.001
	1	6 (60)	6 (19.4)	0 (0)	
	2	4 (40)	16 (51.6)	3 (15.8)	
	3	0 (0)	9 (29)	16 (84.2)	
**Left inguinal LN**	0	1 (10)	3 (9.7)	0 (0)	0.001
	1	9 (90)	28 (90.3)	13 (68.4)	
	2	0 (0)	0 (0)	6 (31.6)	
	3	0 (0)	0 (0)	0 (0)	
**Right inguinal LN**	0	2 (20)	2 (6.5)	0 (0)	0.071
	1	8 (80)	28 (90.3)	17 (89.5)	
	2	0 (0)	1 (3.2)	2 (10.5)	
	3	0 (0)	0 (0)	0 (0)	
**Left popliteal LN**	0	0 (0)	0 (0)	0 (0)	<0.001
	1	7 (70)	16 (51.6)	2 (10.5)	
	2	3 (30)	12 (38.7)	9 (47.4)	
	3	0 (0)	3 (9.7)	8 (42.1)	
Right popliteal LN*	0	0 (0)	0 (0)	0 (0)	<0.001
	1	7 (70)	13 (41.9)	2 (10.5)	
	2	2 (20)	14 (45.2)	8 (42.1)	
	3	0 (0)	4 (12.9)	9 (47.4)	
**Lung**	1	10 (100)	19 (61.3)	4 (21.1)	<0.001^chi^
	2	0 (0)	12 (38.7)	15 (78.9)	

Given is the absolute number (n) and proportion (%) of individuals in each scoring category among the three age classes: Young (n = 10; except RPOP (n = 9)); adult (n = 31); old (n = 19).

^KWT^ = Kruskal-Wallis Test. ^chi^ = χ^2^-test

*young: n = 9. LN = lymph node

### Qualitative assessment

No collagen deposition or fibrous tissue associated with the presence of SLMs was present on Massons Trichrome stains in any of the tissues examined (Figs 2C and 3). Single SLMs were present within the dermis of foot pad sections of all four badgers, for which these tissues were examined.

### Validation of scoring system

**Fig 8:**
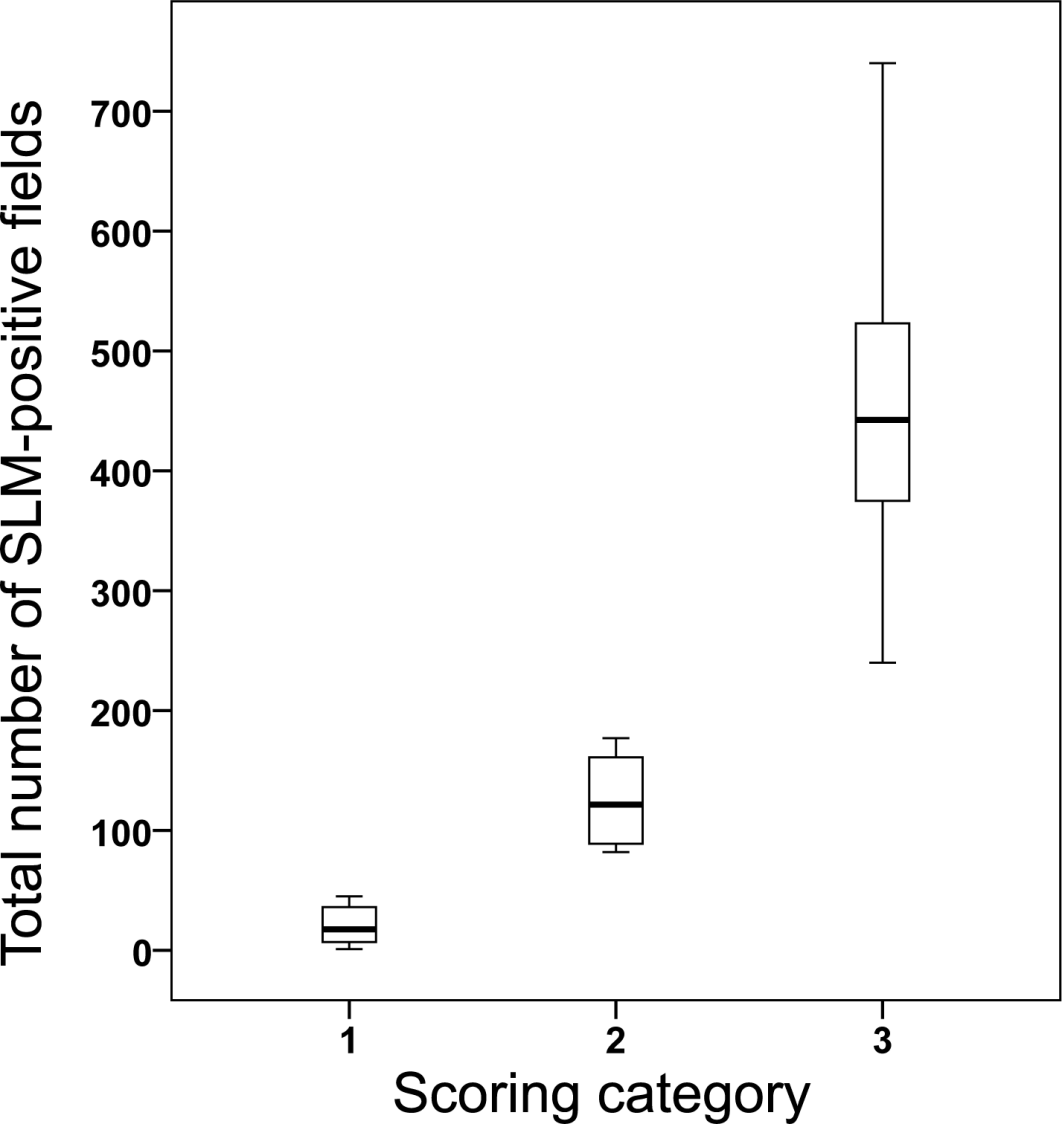
Validation of badger lymph node scoring categories. Boxplots represent the total number of silica-laden macrophage (SLM)-positive fields of lymph nodes within each scoring category 1–3 (see Fig 1). 10 slides were examined per category, and 1000 fields assessed per slide. A positive field was defined as one that contained at least one SLM. One outlier has been omitted from this graph (Scoring category 3: 867).

Lymph node scoring categories were found to be distinct from each other, with positive fields/1000 ranging from 1 to 45 (score 1), 82 to 177 (score 2), and 240 to 867 (score 3), respectively, across 10 tissues assessed for each of the scoring categories (Fig 8, S1 Table). Intra-observer reliability for scoring of lung tissue was strong (Cohens Kappa: 0.8 (95% C.I. = 0.64 to 0.95).

## Discussion

The clinicopathological impact of inhaled silica dusts on the lung and draining lymph nodes has been intensively studied in the case of human silicosis, while the potential health effect of non-occupational exposure to similar environmental dusts is receiving increased attention in the face of climate change, desertification and globally increasing dust levels [34–36].

This study is the first to comprehensively document and quantify ubiquitous and progressive accumulation of SLMs in any mammalian species, where there is no attendant inflammation (e.g. silicosis).

The absence of attendant inflammation in all individuals and tissues examined is a key finding despite the widespread tissue distribution of SLMs and the fact that SLM scores increase with animal age. We were not able to show an association between the degree of SLM accumulation and *M*. *bovis* infection status.

It is plausible to hypothesize, that evolutionary adaption of the badger to a subterranean lifestyle contributes to its tolerance of environmental dust inhalation. Further, a recent in vitro study demonstrated, that badger macrophages fail to produce nitric oxide (NO) [37]. This free radical is an important anti-mycobacterial effector molecule, and was previously shown to be upregulated in an in vivo rat model of silica inhalation [38]. NO is also a driver of macrophage apoptosis, which in turn constitutes a critical step in the pathogenesis of silicosis [10, 11, 39, 40]. Besides rendering the individual more susceptible to mycobacterial infection, a lack of NO production may thus also dampen the badgers’ immune response to environmental dust inhalation. On the other hand, SLM accumulations lacking attendant inflammation, as seen in our study, are not a unique feature of the badger, or subterranean species, for that matter. Similar observations have been made in a wide range of species living aboveground in dry environments, including humans and birds [24–26]. In a large study on mammalian and avian species kept in an arid climate zoo, Brambilla et al. found no correlation between the preferred habitat of a species and the amount of mineral dust within their lungs [24]. Moreover, findings in humans ranging from subclinical simple SLM accumulations in people living in desert climates [25, 26] to severe pulmonary silicosis in mine workers [2] highlight, how the same species may react very differently to the inhalation of siliceous dust.

As mentioned above, a critical step in the pathogenesis of silicosis is silica-induced apoptosis of macrophages [10, 11, 39]: While silica particle uptake was the same in two macrophage populations in an in vivo mouse model of silicosis, only those cells undergoing apoptosis triggered a proinflammatory stimulus [39]. Furthermore, both in vitro and in vivo studies have demonstrated that the surface properties of silica particles modulate their pathogenicity [41]. Specifically, the weathering of particle surfaces, as prevalent in environmental mineral dust, renders silica particles less cytotoxic than those freshly fractured, common in cases of occupational exposure [42–45]. Summarizing the literature, surface properties of inhaled particles rather than host characteristics appear to play a key role in determining whether silicosis ensues following siliceous dust inhalation.

Based on our findings, we therefore suggest that, in contrast to freshly fractured and highly cytotoxic silica and silicates classically involved in cases of occupational silicosis, badgers are typically exposed to relatively non-pathogenic weathered or aged environmental silica and silicates. Thus, chronic mineral dust inhalation constitutes an unavoidable but clinically inconsequential challenge to the badger given its natural habitat.

The pathogenicity of different silica polymorphs, which have the same chemical composition but are arranged in differing crystalline structures, and silicates may also be a contributing factor, with tridymite and crystobalite reported to be more cytotoxic and fibrogenic than stishovite and mica, for example [2, 20]. It was not possible to determine the exact mineralogical composition of the crystalline particles in this study. The majority of the badgers examined were derived from two counties in Ireland (Wicklow (39/60); Mayo (18/60)), while further animals were from county Roscommon. Exact sett locations from which badgers were sourced were not available, but all regions feature diverse soil types, including sands, loam and peat [46]. Typically, badgers preferentially choose sandy soils, which are naturally high in silica and silicates, to establish their setts, as these facilitate burrowing and drain easily [47, 48].

In human medicine, silicosis is a well-established and strong risk factor for tuberculosis [14–17]. This association has been linked to different aspects of the inflammatory response, which ultimately serves to contain inhaled silica particles locally within the lungs and draining lymph nodes [19]. It has been suggested that silica inhalation is a risk factor for *M*. *bovis* infection in the badger [28]. Our findings, specifically the absence of SLM-associated inflammation, and the apparent unhindered movement of SLMs throughout the body reflect fundamental differences in the pathogenesis of silicosis vs. benign SLM accumulations, as discussed above. Therefore, drawing parallels between the situations pertaining in humans and badgers regarding possible immune modulatory effects of inhaled siliceous dust is not appropriate. This is further supported by our finding that *M*. *bovis* infection positive and negative badgers had similar SLM distribution and burdens.

A limitation of the statistical analysis was the small sample size, which resulted in low power when comparing SLM score distributions of *M*. *bovis* positive and negative badgers as confirmed by a post hoc power analysis. Therefore, we cannot rule out a possible bias towards the null hypothesis in this comparison. We must also be mindful that our cross-sectional approach limited our ability to comment directly on cause and effect.

Our study shows that the amount of SLMs in lymphatic tissues is positively associated with the amount of SLMs within the lungs, indicating that the badger shares the respiratory route as the main route of silica and silicate entry with other mammals, including humans. While high SLM scores within intrathoracic lymph nodes that immediately drain the lungs were expected (i.e. tracheobronchial and mediastinal lymph nodes), SLMs were also frequently present in peripheral lymph nodes. Previously, silica and silicates in extrathoracic sites had only occasionally been reported in humans and other mammals [6, 7, 49]. An exception to the presence of large amounts of SLMs within intrathoracic lymph nodes was the anterior mediastinal lymph node, which did not contain SLMs in the majority of badgers. This finding is consistent with the tributary region of this lymph node, which primarily drains the heart, trachea and oesophagus, and not the lungs [50].

High SLM scores in axillary and popliteal lymph nodes were also an unexpected finding. We therefore examined a small number of badger foot pads to investigate a possible percutaneous entry route of silica and silicates, and found single SLMs within the dermis. While inorganic particles may enter the body via microlesions of the skin, our findings, while acknowledging the small sample, do suggest, that this occurs infrequently or to only a small degree. However, a cumulative effect over time cannot be ruled out. Experimental studies in rats also indicate that silica does not readily cross intact mucosal barriers [51]. In our study, very few SLMs were present in the tonsils of just one individual, and the mesenteric lymph nodes of just three individuals, indicating that the alimentary tract is unlikely to be a significant source of silica and silicates entering the lymphatic system.

Whether silica is distributed or redistributed throughout the body via the hematogenous or lymphatic route has been a matter of longstanding debate [5, 22, 49, 52]. In cases of human systemic silicosis, the liver and spleen are commonly affected [5]. Our finding of ubiquitous SLMs in peripheral lymphatic sites, positively associated with lung SLM score, in the absence of SLMs in liver and spleen, suggest the lymphatic route as the more likely pathway for the systemic distribution of SLMs in the badger. This hypothesis challenges our current understanding of the lymphatic system as a ‘one way street’ [5, 22, 49, 52] and highlights the need for a more detailed characterization of the structure and function of the lymphatic system in the badger and other species. We must also be cognizant, however, of the limitations of our study in terms of inferences drawn from histological samples providing a single ‘snap-shot’ in time of the processes occurring.

Finally, previous evidence of progressive accumulation of silica and silicates within tissues over an individual’s life or exposure time, respectively, in both animal species and humans [4, 15, 24], was confirmed by our finding of a positive correlation between SLM tissue score and badger age.

## Conclusion

This study is the first to comprehensively document and quantify the scale of systemic dissemination of inhaled environmental dust in animals or man. Contrary to occupational exposure to silica and silicates in humans, chronic environmental dust inhalation does not cause fibrosing lung disease (e.g. silicosis) in the badger, and we were not able to demonstrate an association between the amount of SLMs and *M*. *bovis* infection status.

## Supporting information

S1 Dataset Study population raw data. Age, sex, and *Mycobacterium bovis* infection status of the badger study population, and silica-laden macrophage scores of each tissue examined.
(XLSX)

S1 Table Validation of lymph node scoring system. Number of positive fields out of 10x100 fields examined per slide. A positive field contained at least one silica-laden macrophage.
(XLSX)

S2 Table Badger characteristics by *M*. *bovis* infection status. (DOCX)
