# Supplementary material for: Environmental dust inhalation in the European badger (Meles meles): Systemic distribution of silica-laden macrophages, pathological changes, and association with Mycobacterium bovis infection status
Source: PLoS One. 2018 Jan 17;13(1):e0190230. doi: 10.1371/journal.pone.0190230 (PMC5771571; doi:10.1371/journal.pone.0190230)
Supplement: S2 Table — (DOCX) [file pone.0190230.s003.docx]

|  | *M. bovis +* (n=30) | *M. bovis* -  (n=30) |
| --- | --- | --- |
| Age |  |  |
| - young | 6 | 4 |
| - adult | 16 | 15 |
| - old | 8 | 11 |
| Geographic origin |  |  |
| - Wicklow | 21 | 18 |
| - Mayo | 8 | 10 |
| - Roscommon | 1 | 2 |
| Sex |  |  |
| - male | 18 | 10 |
| - female | 12 | 20 |
